# A rare case of pediatric primary central nervous system differentiating neuroblastoma: an unusual and rare intracranial primitive neuroectodermal tumor (a case report)

**DOI:** 10.11604/pamj.2021.40.33.30587

**Published:** 2021-09-13

**Authors:** Mehdi Borni, Mohamed Znazen, Najiba Mdhaffar, Mohamed Zaher Boudawara

**Affiliations:** 1 Department of Neurosurgery, University Hospital Center Habib Bourguiba, Sfax, Tunisia,; 2 Department of General Medicine, Regional Hospital of Jebeniana, Sfax, Tunisia

**Keywords:** Neuroblastoma, surgery, radiotherapy, chemotherapy, case report

## Abstract

Neuroblastoma represents the most common solid extracranial tumor in children under 5, accounting for 8% to 10% of all childhood cancers. Primary central nervous system (CNS) neuroblastomas are a very rare location and only few cases are available in the literature. It was first described in 1973 by Hart and Earl as supratentorial primitive neuroectodermal tumors. Clinical presentation is highly variable and depends on the initial location of the tumor. Regarding imaging, primary brain neuroblastoma shows no pathognomonic appearance on brain computed tomography (CT) whether or not enhanced or magnetic resonance imaging (MRI). There were no standard guidelines available for the adjuvant treatment in case of primary CNS neuroblastoma. Surgery remains the main and the first tool toward these lesions. Radiotherapy associated or not to chemotherapy is offered based on patient´s age. Here, the authors report a new pediatric case of primitive central nervous system neuroblastoma revealed by an intracranial hypertension syndrome and confirmed by both histopathological and immunohistochemistry study after a gross total surgical excision. The postoperative course was uneventful and the child had good recovery.

## Introduction

Neuroblastoma represents the most common solid extracranial tumor in children under 5, accounting for 8% to 10% of all childhood cancers [1]. Much of the literature is available mainly on the primary neuroblastoma of abdomen with its staging system, treatment and prognosis. Primary central nervous system (CNS) neuroblastomas are an extremely rare location and only few cases were reported previously. It was first described in 1973 by Hart and Earl as supratentorial primitive neuroectodermal tumors. The literature remains sparse when it comes to this type of tumor. Here, the authors report through this report a new pediatric case of primitive central nervous system neuroblastoma revealed by an intracranial hypertension syndrome, screened by imaging and confirmed by both histopathological and immunohistochemistry study after a gross total surgical excision.

## Patient and observation

**Patient information:** a 6-year-old boy with medical history of mild persistent asthma since the age of one year undergoing long-term control medications made of inhaled corticosteroids and who is followed in the department of child psychiatry for attention deficit hyperactivity disorder under psychological counseling was admitted in our department of neurosurgery for raised intracranial pressure of 6-month duration made of intractable holocranial throbbing headache with several episodes of vomiting and bilateral blurred vision outside any context of head trauma of associating fever.

**Clinical findings:** upon neurological examination, he was perfectly conscious, a little agitated without any motor or sensory deficit or other localization signs. Examination of the fundus revealed a bilateral grade I papilledema. The rest of general physical examination of the patient did not show any abnormalities.

**Figure 1:**
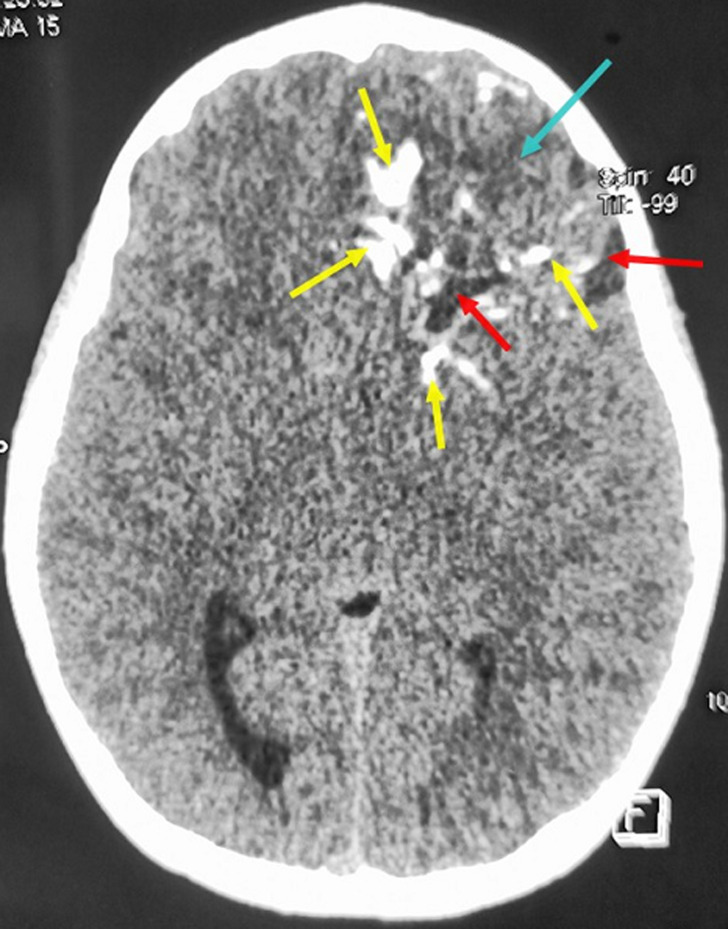
axial non-enhanced brain CT scan showing a left basifrontal tumor measuring 50 x 45 mm in diameter reaching the ipsilateral temporal lobe; this mass had a triple fleshy component (blue arrow), cystic (red arrows) and scattered foci of calcification (yellow arrows) in variable proportions; note the midline shift estimated at 6 mm without any uncal herniation

**Figure 2:**
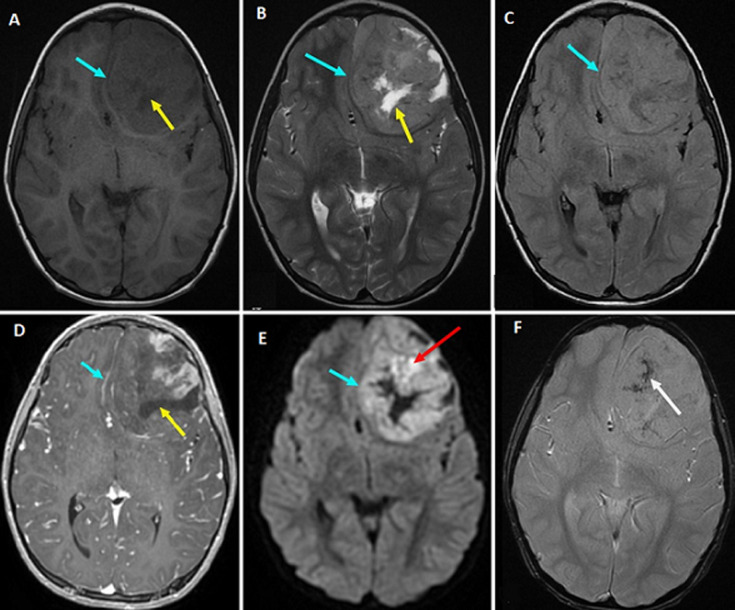
A) brain MRI in axial section showing a left basifrontal intra-axial lesion extended to the left polar temporal region, in hypointense on the T1-weighted sequences; B) in heterogeneous isosignal on the T2 sequences; C) not surrounded by an edematous reaction on FLAIR (fluid attenuated inversion recovery) sequence; D) enhancement after gadolinium injection is heterogeneous; E) note the heterogeneous hyper signal on the diffusion weighted images (DWI) without any hyperperfusion on perfusion sequences; F) cystic plaques and hypointense spots are seen on the gradient echo sequence; there was no intra-tumor bleeding on the susceptibility sequences

**Diagnostic assessment:** a non-enhanced brain CT scan (Figure 1) was performed immediately. It has revealed a left basifrontal mass measuring 50 x 45 mm in diameter, which extends to the ipsilateral temporal lobe. This lesion has a triple fleshy component, cystic and scattered calcifications in variable proportions. The assembly exerts a midline shift estimated at 6 mm without uncal herniation. Brain MRI (Figure 2) confirmed the presence of a left basifrontal intra-axial lesion extended to the left polar temporal region, in hypointense on T1-weighted sequences and in heterogeneous isosignal on T2 sequences not surrounded by an edematous reaction on fluid attenuated inversion recovery (FLAIR) sequence. This mass contains cystic plaques and hypointense spots on the gradient echo sequence. The lesion was enhanced heterogeneously after gadolinium injection. It is associated with a heterogeneous hyper signal on the diffusion weighted images without any hyperperfusion on perfusion sequences. There was no intra-tumor bleeding on the susceptibility sequences. The complete blood count was normal (white blood cells: 17400 cells per microliter (cells/mcL); red blood cells: 3.09 million cells/mcL; haemoglobin: 9 grams per deciliter (gm/dL); hematocrit: 26.3%; and platelets: 337,000 platelets/mcL). Assessment of blood electrolytes levels was in normal range. The chest radiograph revealed no abnormalities. The patient's hemodynamic status was stable.

**Figure 3:**
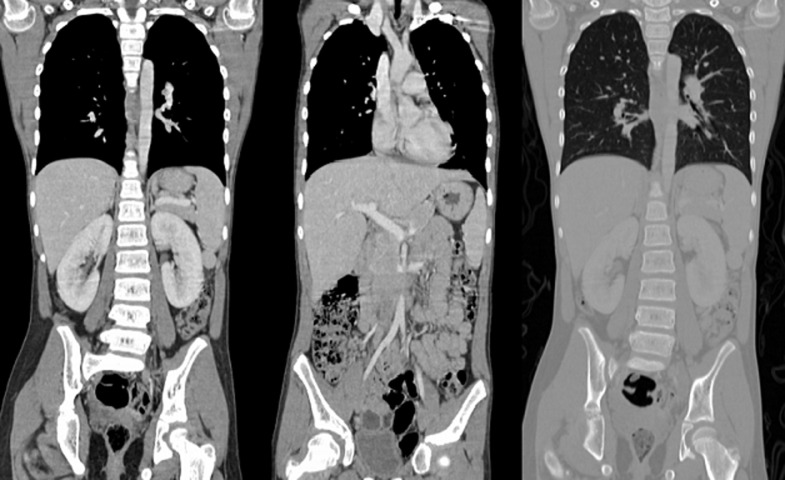
a thoraco-abdomino-pelvic CT scan in several coronal sections showing the absence of a primary lesion or any unusual enhancing mass

**Figure 4:**
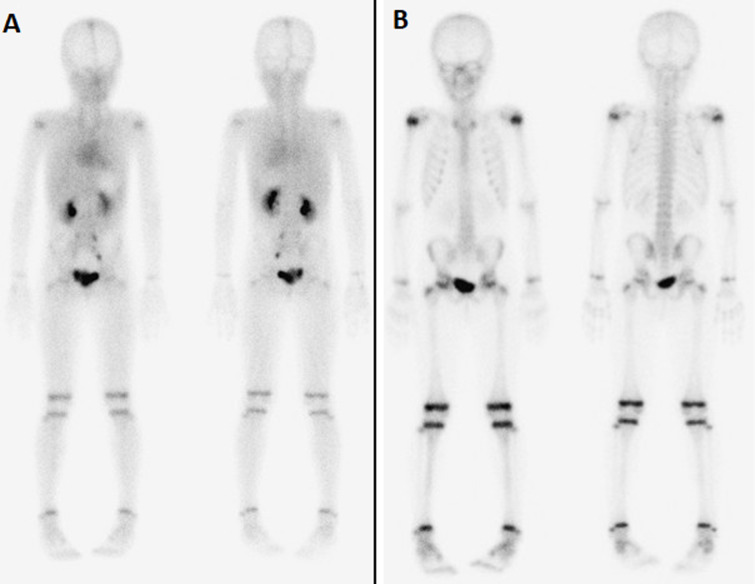
planar whole-body skeletal scan in anterior and posterior projections showing the absence of unusual increased 99mTc-MDP uptake in the whole skeleton; A) anterior and posterior blood pool whole body views; B) delayed anterior and posterior whole body bone scan

As part of the extension assessment, a thoraco-abdomino-pelvic computed tomography (CT) scan (Figure 3) was performed which failed to show any evidence of primary lesion or enhancing mass. The whole body skeletal scintigraphy confirms the absence of any unusual increased tracer (technetium 99m-methyl diphosphonate: ^99m^Tc-MDP) uptake in the whole skeleton (Figure 4). We did not perform the I-123 MIBG (meta-iodobenzylguanidine) scintigraphy for lack of means.

**Therapeutic intervention:** the patient underwent, through a frontotemporal craniotomy in supine position with his head rotated about 30° contralaterally and placed in a standard Mayfield skull clamp, a gross total excision of his tumor having, preoperatively, a firm and vascular appearance without definite plane with normal brain parenchyma. Hemostasis was laborious, the dura was tightly closed and the bone flap was returned and secured with four transosseous stitches.

**Figure 5:**
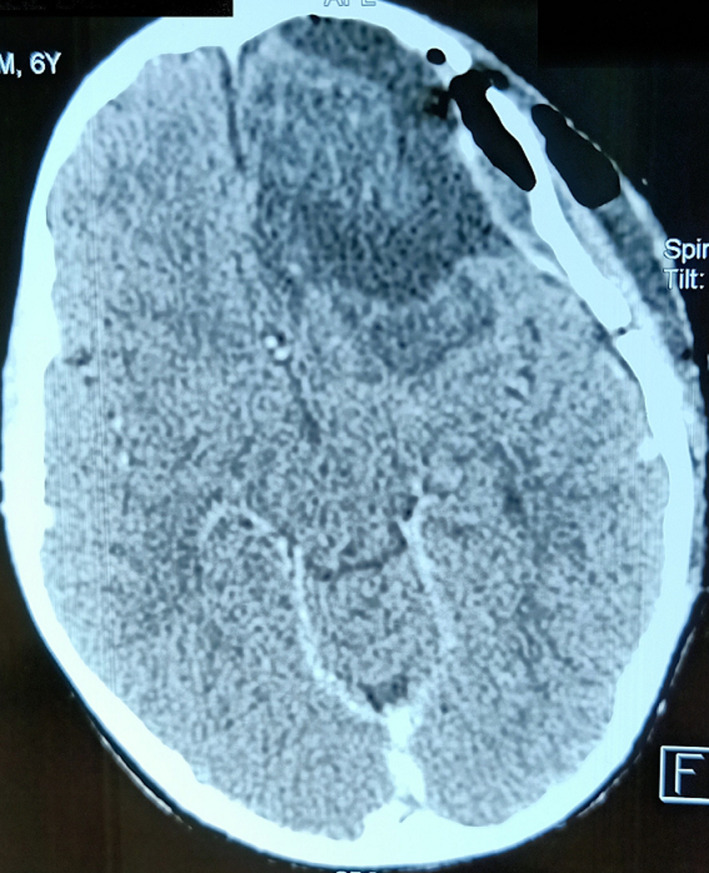
axial non-enhanced postoperative CT scan showing a porencephalic cavity at the level of the tumor bed associated with pneumocephalus even subcutaneously; note the disappearance of midline shift and good decompression of healthy parenchyma

**Follow-up and outcomes:** the postoperative awakening was uneventful when general sedation was gradually stopped. Postoperative CT scan at day 2 (Figure 5) showed a porencephalic cavity at the level of the tumor bed associated with pneumocephalus even subcutaneously and disappearance of the midline shift and good decompression of healthy parenchyma.

**Figure 6:**
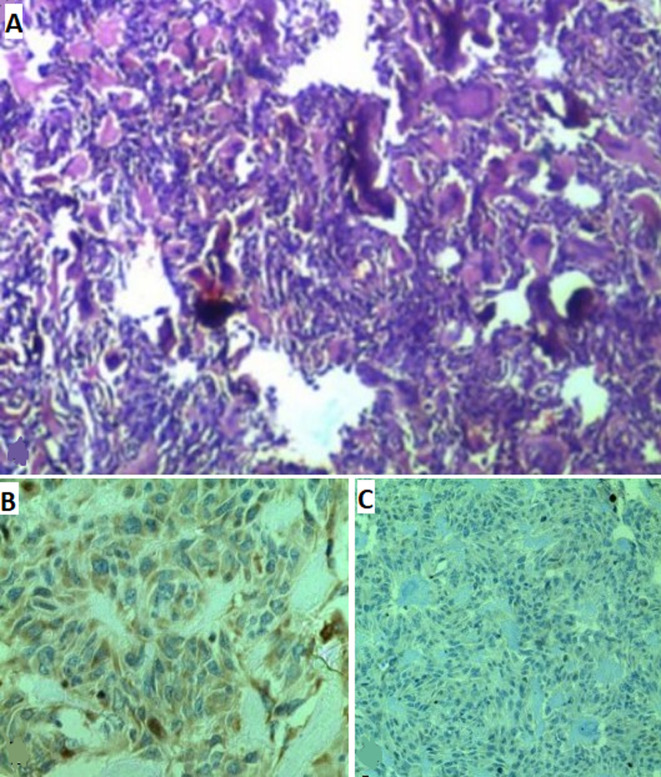
A) microscopic histopathological images showing a focal necrosis tumor proliferation, vaguely lobulated and crossed by fibrous septa (H & E 400x); this proliferation is of moderate cellularity made up essentially of small round monomorphic basophilic cells with a rounded or oval nucleus with a high nuclear/cytoplasmic ratio; focally, there was a transition between these undifferentiated embryonic cells with other neurocytically differentiated cells; the background of the proliferation is clearly fibrillary with presence of numerous Homer-Wright rosette images and pseudo-rosettes (H & E 400x); it is associated with micro calcifications and rare spumous histiocytes; B) microscopic immunohistochemistry (IHC) study showing that tumor cells were positive for INI-1, neurospecific enolase (NSE) (IHC for NSE 400x); C) synaptophysin (IHC for synaptophysin 400x)

Histopathological examination (Figure 6A) showed a focal necrosis tumor proliferation, vaguely lobulated and crossed by fibrovascular septa. This proliferation is of moderate cellularity made up essentially of small round monomorphic basophilic cells with a rounded or oval nucleus with a high nuclear/cytoplasmic ratio. Mitosis was rare. Focally, there was a transition between these undifferentiated embryonic cells with other neurocytically differentiated cells. The background of the proliferation is clearly fibrillary with presence of numerous Homer-Wright rosette images and pseudo-rosettes. It is associated with micro calcifications and rare spumous histiocytes. The immunohistochemistry study was carried out on paraffin sections. Tumor cells were positive for INI-1, neurospecific enolase (NSE) (Figure 6B), Synaptophysin (Figure 6C), and CD 56 antibodies and were negative for epithelial membrane antigen (EMA), PS100, glial fibrillary acidic protein (GFAP), neurofilament and desmin. The Ki 67 proliferation index is estimated at 40%. All these features were in favor of cerebral differentiating neuroblastoma. The postoperative course was uneventful and the child had good recovery with a clear reduction in headaches. He was discharged from hospital at day 4 postoperatively, without any neurological signs under prophylactic antiepileptic drug based on sodium valproate at a dose of 30 mg/kg/day in 3 divided doses.

**Patient perspective:** during hospital stay and at discharge, the patient´s mother was delighted with the care.

**Informed consent:** the patient´s mother was informed about the report, why this case was peculiar and the authors interest in publishing it. She gave informed consent to allow the authors to use her case for this case report.

**Patient's consent:** informed consent was obtained from the patient´s parent for us to use the case.

## Discussion

Neuroblastoma is the most common solid extracranial tumor in children under 5, accounting for 8% to 10% of all childhood cancers [1]. Its prevalence is approximately 1 case per 7,000 births [2]. Several studies have indicated that this incidence is fairly uniform around the world, at least in industrialized countries [1,2]. This tumor is almost exclusively a pediatric neoplasia and is the most common cancer in the neonatal period [3]. A few studies have attempted to establish a relationship between this tumor and certain intrauterine exposures, but such a relationship could not be demonstrated and confirmed in larger cohorts [4]. Factors that have been investigated include occupation, that is, exposure to chemicals in specific industries, smoking, drinking, drug use during pregnancy and birth factors; however, the results have been inconsistent [5].

Primary central nervous system (CNS) neuroblastomas are a very rare location and few cases are available in the literature. It was first described in 1973 by Hart and Earl as supratentorial primitive neuroectodermal tumors. They are extremely variegated in histology. Primary CNS localization has often been classified as part of primary neuroectodermal tumors, although this is controversial. The diagnosis is confirmed by a surgical pathologist. On microscopy, tumor cells are usually described as small, rounded, bluish cells with rosette patterns (Homer-Wright pseudorosettes). A variety of immunohistochemical staining is used for histologic distinction of neuroblastomas from other similar pathologies such as rhabdomyosarcoma, Ewing's sarcoma, lymphoma and Wilms tumor. All these features were also suggested by Horten and Rubinstein [6]. On electron microscopy, the presence of neurosecretory granules helped to confirm the diagnosis [7]. In our case, Homer-Wright pseudorosettes were present associated to transition between the undifferentiated embryonic cells with other neurocytically differentiated cells. The Ki 67 proliferation index was estimated at 40% testifying to the aggressiveness of this neuroblastoma.

Clinical presentation is highly variable and depends on the initial location of the tumor, the size, as well as the local and distant extension [1]. However, there are rare but characteristic symptoms that are very suggestive of neuroblastoma. These manifestations include: paralysis of the lower limb related to the intraspinal epidural extension of a primary para-spinal tumor (4%); severe diarrhea which does not respond to standard treatment and which is due to the production of vasoactive intestinal peptide (VIP) by tumor cells (4%); acute cerebellar encephalopathy (2-8%); Claude Bernard Horner syndrome in patients with lesions of the upper thoracic or cervical sympathetic ganglia (1-7%); arterial hypertension, flushing and occasional periods of excessive sweating caused by an increase in the concentration of catecholamines (0-2%) [1]. However, this clinical presentation is still non-specific concerning the CNS primary locations and cannot be distinguished from other aggressive intracranial space-occupying lesions. It includes symptoms of raised intracranial pressure, seizures and focal neurological deficit. This is concordant with our patient who only experienced signs of intracranial hypertension made of headaches, vomiting and bilateral blurred vision with no others signs of distant extension.

Regarding imaging, primary brain neuroblastoma shows no pathognomonic appearance on brain computed tomography (CT) whether or not enhanced or magnetic resonance imaging (MRI). Cystic or calcium formations can be observed in some patients accompanying the fleshy component which remains predominant. This feature agrees with our patient´s imaging with no particular radiological findings. However, exact data for the same is not available in the literature due to the rarity of the disease [8]. Investigation must be completed by a thoraco-abdomino-pelvic CT scan and I-123 meta-iodobenzylguanidine (MIBG) scintigraphy. The mechanism is that MIBG is taken up by sympathetic neurons in a function analogous to the neurotransmitter norepinephrine. In recent years it has become possible to attach a radioactive emitter, the radioactive isotope of iodine I-131, to MIBG. This “radio-labelled” MIBG can be used in two ways in the care of children with neuroblastomas. When it is radio-ionized with I-131 or I-123 (another radioactive isotope of iodine), it becomes tremendously useful for the diagnosis and monitoring of the response to treatment of this disease, mainly in the detection of secondary cancers, metastases [9]. In our patient we performed only a thoraco-abdomino-pelvic CT scan. We did not perform the I-123 MIBG scintigraphy for lack of means.

There were no standard guidelines available for the adjuvant treatment in case of primary CNS neuroblastoma. Surgery remains the main and the first tool toward these lesions. Radiotherapy associated or not to chemotherapy is offered based on patient´s age. In their study, Bennett *et al*. [10] suggested that prophylactic craniospinal irradiation is probably justified because of the high incidence of local recurrence and the relative frequency of distant cerebrospinal metastases. However, Berger *et al*. [11] stated that spinal radiotherapy should only be reserved for people with obvious signs of cerebrospinal fluid metastasis or myelogram abnormalities. The use of chemotherapeutic agents used in combination has been shown to be effective against neuroblastoma. The agents most used in the induction and conditioning of stem cell transplantation are composed of platinum, such as cisplatin, carboplatin), alkylating agents (cyclophosphamide, ifosfamide, melphalan), topoisomerase II inhibitor (etoposide), antibiotics derived from anthracycline (doxorubicin) and V/inca alkaloids (vincristine). Some regimens that include new topoisomerase I inhibitors (topotecan and irinotecan) at induction have also been found to be effective against recurrent disease.

With current treatments, patients with intermediate and low risk of the disease tend to have a very good prognosis, with cure rates higher than 90% in low-risk patients and 70%-90% in intermediate-risk patients [12]. In contrast, in the past two decades, therapy for high-risk neuroblastoma patients has resulted in cures of only about 30%. The addition of antibody therapy has dramatically increased survival rates in patients at high risk for the disease. In March 2009, an analysis by a children's oncology group that studied 226 high-risk patients showed that the 2-year disease-free survival was 66% in the group that randomly received antibodies to chromosome 14.18 with GM-CSF and IL-2 and 46% in the group that did not receive the antibody. Randomization was stopped for all patients so that they could all enroll in the process and receive the antibody treatment.

Concerning prevention and screening, several attempts have been made to screen neuroblastomas by methods for detecting catecholamine metabolites in urine samples; screening programs since the 1980s have included 3-week-old newborns in Japan, 3-week-olds and 6-month-olds in Canada and 1-year-olds in Germany [13,14]. Japan began screening neuroblastoma in six-month-old infants by testing the concentration of homovanillic acid (HVA) and vanillin-mandelic acid (VMA) in the urine in 1984. That screening does not reduce the risk of death from neuroblastoma, but poses a risk of diagnosing a cancer that may spontaneously regress and unnecessarily expose children to surgery and chemotherapy [15-17]. A large Canadian study found that pregnant women who took folic acid enriched vitamin kits before and during the first 3 months of pregnancy had a 60% reduced risk of developing neuroblastoma in their babies [18].

## Conclusion

Primary CNS neuroblastoma remains a rare pathology with high risks of local or distant recurrence. It carries a severe prognosis due to its aggressive nature. The preoperative diagnosis is based on imaging although it is quite difficult. It should be considered as one of the differential diagnoses of tumors in the pediatric population. Radical surgery followed by adjuvant chemotherapy and/or radiotherapy appears to be the best treatment at present.
